# Brief Alcohol Interventions are Effective through 6 Months: Findings from Marginalized Zero-inflated Poisson and Negative Binomial Models in a Two-step IPD Meta-analysis

**DOI:** 10.1007/s11121-022-01420-1

**Published:** 2022-08-17

**Authors:** Eun-Young Mun, Zhengyang Zhou, David Huh, Lin Tan, Dateng Li, Emily E. Tanner-Smith, Scott T. Walters, Mary E. Larimer

**Affiliations:** 1 Department of Health Behavior and Health Systems, School of Public Health, University of North Texas Health Science Center, 3500 Camp Bowie Blvd., Fort Worth, TX 76107 USA; 2 Department of Biostatistics and Epidemiology, University of North Texas Health Science Center, Fort Worth, TX 76107 USA; 3 School of Social Work, University of Washington, Seattle, WA 98195 USA; 4 121 Westmoreland Ave, White Plains, NY 10606 USA; 5 Department of Counseling Psychology and Human Services, University of Oregon, Eugene, OR 97403 USA; 6 Department of Psychiatry and Behavioral Sciences, University of Washington, Seattle, WA 98195 USA

**Keywords:** Brief alcohol intervention, Brief motivational intervention, College students, Individual participant data, IPD, Meta-analysis, Integrative data analysis

## Abstract

To evaluate and optimize brief alcohol interventions (BAIs), it is critical to have a credible overall effect size estimate as a benchmark. Estimating such an effect size has been challenging because alcohol outcomes often represent responses from a mixture of individuals: those at high risk for alcohol misuse, occasional nondrinkers, and abstainers. Moreover, some BAIs exclusively focus on heavy drinkers, whereas others take a universal prevention approach. Depending on sample characteristics, the outcome distribution might have many zeros or very few zeros and overdispersion; consequently, the most appropriate statistical model may differ across studies. We synthesized individual participant data (IPD) from 19 studies in Project INTEGRATE (Mun et al., 2015b) that randomly allocated participants to intervention and control groups (*N* = 7,704 participants, 38.4% men, 74.7% White, 58.5% first-year students). We sequentially estimated marginalized zero-inflated Poisson (Long et al., 2014) or negative binomial regression models to obtain covariate-adjusted, study-specific intervention effect estimates in the first step, which were subsequently combined in a random-effects meta-analysis model in the second step. BAIs produced a statistically significant 8% advantage in the mean number of drinks at both 1–3 months (*RR* = 0.92, 95% *CI* = [0.85, 0.98]) and 6 months (*RR* = 0.92, 95% *CI* = [0.85, 0.99]) compared to controls. At 9–12 months, there was no statistically significant difference in the mean number of drinks between BAIs and controls. In conclusion, BAIs are effective at reducing the mean number of drinks through at least 6 months post intervention. IPD can play a critical role in deriving findings that could not be obtained in original individual studies or standard aggregate data meta-analyses.

**Supplementary Information:**

The online version contains supplementary material available at 10.1007/s11121-022-01420-1.

## Introduction

The improved utilization of existing individual participant data (IPD) has emerged as a major driver of innovation for scientific discoveries. Pooling IPD via integrative data analysis (IDA; Curran & Hussong, 2009) or IPD meta-analysis (Riley et al., 2010; Sutton et al., 2008) can enable investigation of new questions that may be difficult or impossible to answer in any individual primary study. Although some differences exist between IDA and IPD meta-analysis methods, we hereafter use the two terms interchangeably because they share similar advantages and challenges (Mun et al., 2015b; Mun & Ray, 2018). In alcohol prevention research, one of the primary advantages of IPD methods is that they can better account for the low base rates of alcohol misuse among adolescents and young adults (Curran et al., 2017), which can be challenging to examine in individual primary research studies. By pooling IPD across multiple studies, intervention effect estimates may become more precise and reliable (Curran & Hussong, 2009), and different types of responders to intervention may be better detected as subgroups (Borenstein & Higgins, 2013; Brown et al., 2013).

Analysis of IPD has well-known challenges, foremost of which is how to account for study-level heterogeneity in measures, designs, and samples. For intervention studies, in particular, the number of intervention groups per study can differ; this can create study-level missing data when pooled, although it may be circumvented by setting study-by-intervention arms as the highest data level and estimating within-study group differences in outcomes (Huh et al., 2015, 2019). Similarly, heterogeneity in intervention content (Mun & Ray, 2018; Ray et al., 2014), study design (Jiao et al., 2020), and measures (Bauer & Hussong, 2009; Curran et al., 2014; Huo et al., 2015; Hussong et al., 2019; Mun et al., 2016, 2019) can be overcome using advanced analytical methods, although there are also limits to these methodological approaches (Hussong et al., 2013; Mun et al., 2015b).

The current study focuses on appropriately analyzing the most common measure of alcohol outcomes, specifically weekly number of drinks, in brief alcohol interventions (BAIs) and overcoming heterogeneity in outcome distributions due to sample differences across studies included in the synthesis. Many alcohol outcomes provide discrete count data that follow nonnormal distributions with excessive zeros and a heavy tail (Atkins et al., 2013; Huh et al., 2019), which may be expected in universal preventive intervention studies (e.g., those targeting all students, regardless of their prior alcohol use). However, very few zeros may also be observed in selective and indicated intervention studies for participants with heavy alcohol misuse (e.g., those studies focused solely on students referred for alcohol policy infractions). Depending on data distributions, the analyses may have to accommodate excessive zeros, little to no zeros, and/or overdispersion (i.e., positive skew) when deriving study-specific and overall intervention effects. In the next section, we describe this issue and the corresponding statistical models in greater detail.

### Poisson, Negative Binomial, Zero-Inflated Poisson, and Marginalized Zero-Inflated Poisson Regression Models

Count distribution-based generalized linear models have gained increasing popularity for modeling count outcomes. Among the distributions for modeling count outcomes, the Poisson regression model has the most straightforward formulation, which models the mean parameter of the outcome and restricts the expected value (i.e., mean) to be equal to its variance. Therefore, when the variance of the distribution is larger than the mean of the distribution (i.e., “overdispersion”), Poisson regression can underestimate variance and yield invalid inference. If the variance is greater than the mean, the negative binomial (NB) model is an alternative count regression model to accommodate this overdispersion. With an additional “dispersion” parameter, NB regression allows variability greater than the mean and flexibility in accommodating overdispersion.

Suppose the primary study sample size is $n$, and for $i$ th subject, $i=\mathrm{1,2},\dots ,n$. The NB model can be formally expressed as:

$${Y}_{i}\sim NB\left({v}_{i},k\right),$$

$$\mathrm{log}\left({v}_{i}\right)={\beta }_{0}^{NB}+{\beta }_{1}^{NB}\cdot Intervention+{\beta }_{p}^{NB}*Additional\ Covariates,$$

where ${v}_{i}=E({Y}_{i})$ is the overall mean of the outcome, $k$ is the dispersion parameter of the NB distribution, ${\beta }_{1}^{NB}$ is the intervention effect on the overall mean for the entire population, and ${\beta }_{p}^{NB}$ is the vector of $p$ covariate effects. A symbol * denotes element-wise multiplication between a vector of covariate effects and a vector of covariate values.

The Poisson and NB regression models, however, do not account for zero inflation, a phenomenon whereby a count outcome has a large proportion of zero values beyond the expected proportion under conventional count models. If excessive zeros are ignored, effect size estimates could be biased and erroneously fail to detect statistical significance (Perumean-Chaney et al., 2013), although that bias may be mathematically corrected in some instances (see Zhou et al., 2021).

The zero-inflated Poisson (ZIP) regression model accounts for excessive zeros by assuming that the count outcome follows a mixture of a Poisson distribution and a point mass at zero (i.e., the structural zeros). For example, when weekly number of drinks is the outcome, the Poisson part corresponds to a subpopulation of participants who may or may not drink at a given assessment, and the structural zero part corresponds to a subpopulation of participants who “predictably” do not drink (i.e., abstainers). Intervention effects are estimated in two parts–the rate ratio (RR) of the mean in the Poisson part (e.g., number of drinks, including some zeros from those who happened not to drink) and the odds ratio (OR) of being a structural zero (e.g., abstainers vs. non-abstainers) in the structural zero part.

When estimating an intervention effect, the ZIP model can be formally presented as follows:

$${Y}_{i}\sim \left\{\begin{array}{c}\begin{array}{cc}0,& \mathrm{with\ probability}\ {\pi}_{i} \end{array}\\ \begin{array}{cc}Poisson\left({\mu}_{i}\right),& \mathrm{with\ probability}\ 1-{\pi}_{i}\end{array}\end{array}\right.,$$

$$\begin{array}{l}\mathrm{log}\left(\frac{{\pi }_{i}}{1-{\pi }_{i}}\right)={\gamma }_{0}^{ZIP}+{\gamma }_{1}^{ZIP}\cdot Intervention+ {\gamma }_{p}^{ZIP}\\\qquad\qquad\qquad*\ Additional\ Covariates,\ \mathrm{and}\end{array}$$

$$\mathrm{log}\left({\mu }_{i}\right)={\beta }_{0}^{ZIP}+{\beta }_{1}^{ZIP}\cdot Intervention+{\beta }_{p}^{ZIP}*Additional\ Covariates,$$

where ${\mu }_{i}=E({Y}_{i}|{Y}_{i}\ \mathrm{from\ the\ Poisson\ part})$ is the mean of the Poisson part in the ZIP model, ${\pi }_{i}=Prob\left({Y}_{i}\ is\ a\ structural\ zero\right)$ is the structural zero rate, ${\gamma }_{1}^{ZIP}$ is the intervention effect on the structural zero part, and ${\beta }_{1}^{ZIP}$ is the intervention effect on the Poisson part. Likewise, ${\gamma }_{p}^{ZIP}$ and ${\beta }_{p}^{ZIP}$ are the vectors of $p$ covariate effects on the structural zero part and the Poisson part, respectively. Under the ZIP model, the intervention effect is separately estimated by ${\gamma }_{1}^{ZIP}$ and ${\beta }_{1}^{ZIP}$, representing intervention effects on two distinct subpopulations: those who do not drink at all (i.e., the structural zero part) and those who may or may not drink (the Poisson part), respectively.

The marginalized ZIP (MZIP) model (Long et al., 2014) is based on the formulation of the ZIP model. Suppose the “overall” mean of the outcome, i.e., ${v}_{i}=E\left({Y}_{i}\right)=(1-{\pi }_{i}){\mu }_{i}$, is of interest. Under the MZIP model, ${v}_{i}$ can be directly modeled through

$$\begin{array}{l}\mathrm{log}\left({v}_{i}\right)={\beta }_{0}^{MZIP}+{\beta}_{1}^{MZIP}\cdot Intervention\\\qquad\qquad\ +\ {\beta }_{p}^{MZIP}*Additional\ Covariates.\end{array}$$

**Fig. 1:**
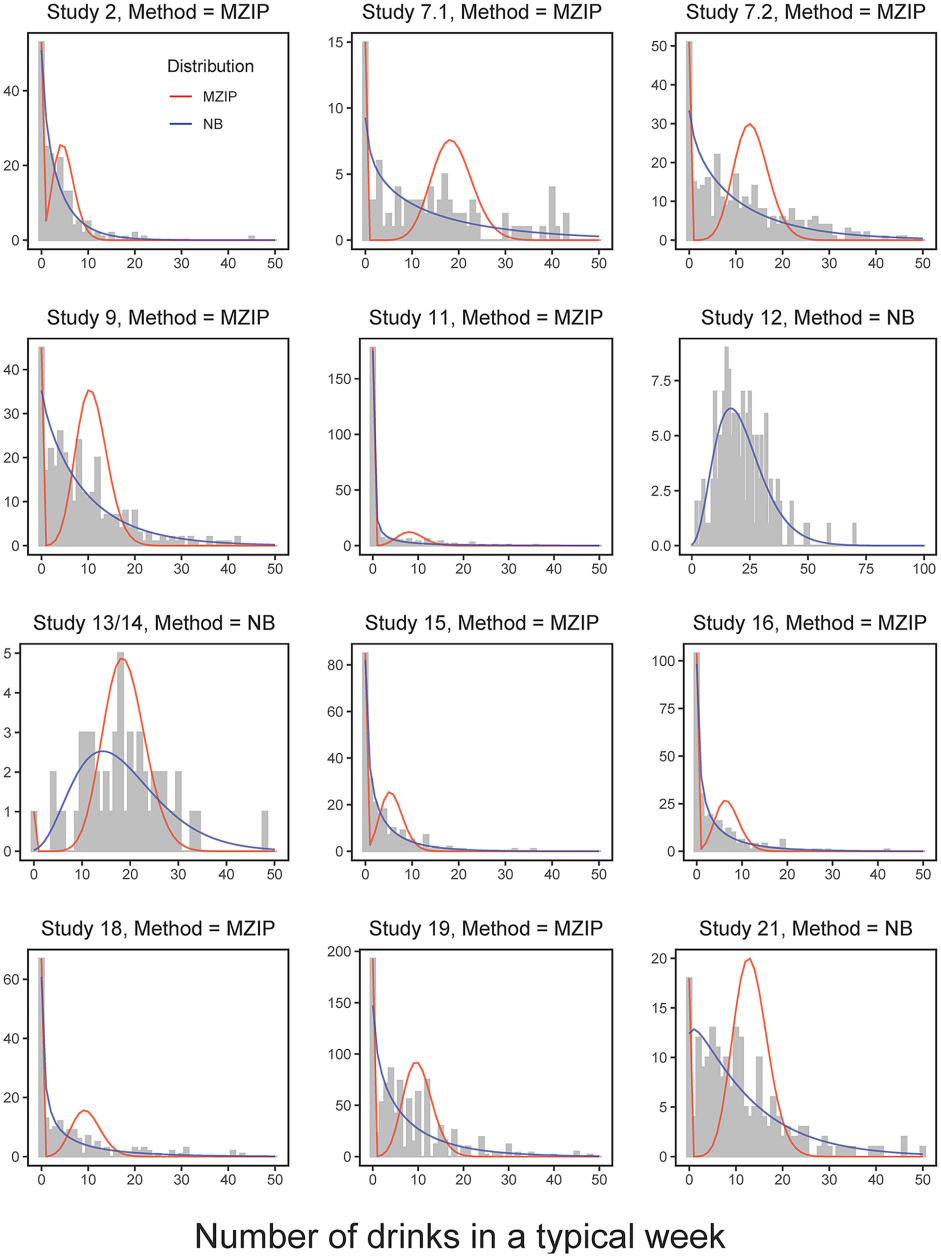
Frequency distributions with theoretical distributions for number of drinks in a typical week. *Note.* Y-axis = Frequency. Outcome distributions at 1–3 months post intervention by study with two predicted probabilities from marginalized zero-inflated Poisson (MZIP) and negative binomial (NB) models. Descriptive statistics are shown in Table 2. MZIP models accommodate excess zeros and peaks of nonzero distributions in most studies. NB models underestimate zeros in many studies (e.g., studies 7.1, 7.2, 9, 19) and miss nonzero peaks in most studies. Study 12 had no zero value. Therefore, study 12 was fitted only with the negative binomial model

These parameters can be estimated by maximizing the likelihood function using nonlinear optimization algorithms. In the MZIP model, the term ${\beta }_{1}^{MZIP}$ quantifies the intervention effect on the overall mean outcome for the entire population, including those with structural zero values for the count outcome. Therefore, estimates from the MZIP model share the same interpretation as those from the NB model. This commensurate interpretation of the intervention effect from the MZIP and NB models makes it attractive when answering questions about whether and to what extent an intervention is effective across the entire population, especially for analysis of IPD from multiple studies. The feature of drawing inference for the entire population from the MZIP model can also help assess whether an intervention is efficacious because there is a single estimate rather than two separate, compartmentalized estimates for the intervention effect of interest. Since the MZIP model accommodates excessive zeros, it can be used along with the NB model in IPD meta-analyses where excessive zeros are present in some studies (see Fig. 1 for heterogeneous outcome distributions) while maintaining the equivalent interpretation of intervention effects across heterogeneous distributions.

### Brief Alcohol Interventions

Since the first launch of the Brief Alcohol Screening and Intervention for College Students (BASICS) in 1990, BAIs focus on personalized feedback provided in the nonjudgmental and supportive style of motivational interviewing (MI; Miller & Rollnick, 1991) have proliferated (Larimer et al., 2021). The initial BASICS programming has been adapted and modified for different populations and settings. While the modifications may be necessary developments to attend to local constraints, their impact on intervention effectiveness and, consequently, its public health impact is unclear (see Larimer et al., 2021 for greater detail). A methodological challenge lies at the heart of this issue. To evaluate and optimize BAIs, it is critical to have a credible overall effect size estimate as a benchmark. Estimating such an effect size benchmark has been challenging because alcohol outcomes often represent responses from a mixture of individuals within the same trial: those at high risk for alcohol misuse, occasional nondrinkers, and abstainers. Moreover, some intervention studies exclusively focus on heavy drinkers or abstainers, whereas others take a universal prevention approach. Such study-level differences give rise to different outcome distributions, which are analyzed differently and sometimes less optimally by original investigators in primary studies.

Most BAIs analyze alcohol count outcomes as if they were normally distributed using analysis of variance or covariance (Tan et al., 2022b). Even when NB or ZIP models are appropriately used, the former produces one intervention effect estimate for the entire population, whereas the latter produces two effect estimates for two subpopulations. Therefore, it is challenging to pool data from multiple studies that may or may not have excessive zeros for synthesis. With IPD from original studies, outcomes can be analyzed appropriately and sequentially for each study and then pooled for synthesis. The current paper is aimed at providing better clarity about BAI effectiveness via the innovative application and demonstration of the MZIP model in combination with the conventional count model (i.e., NB) to produce a unified “overall” intervention effect estimate that accommodates studies with and without excessive zeros.

## Methods

### Participants

**Fig. 2:**
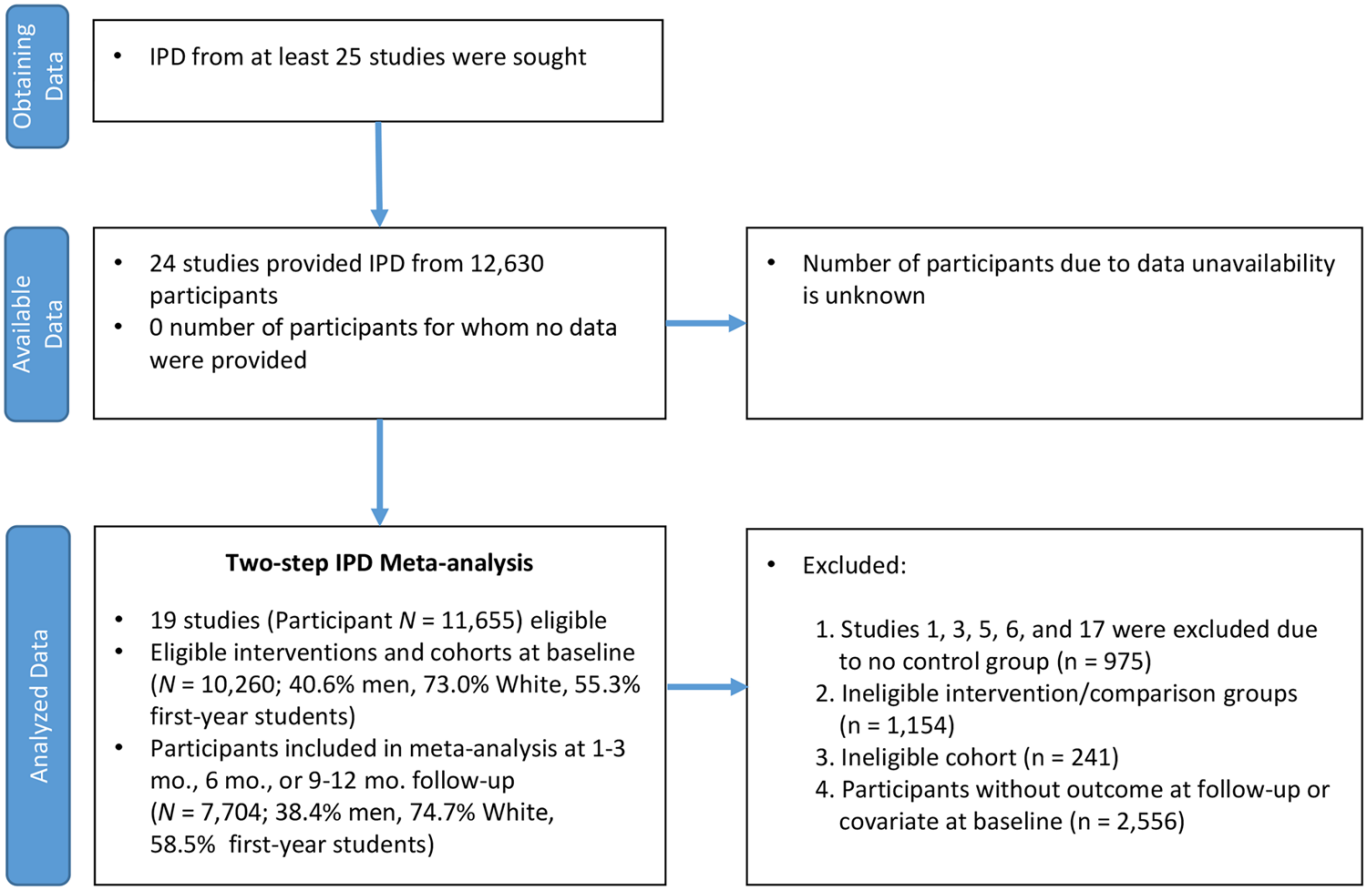
PRISMA flow diagram for individual participant data

The data come from Project INTEGRATE (Mun et al., 2015b), an ongoing large-scale synthesis study aimed at examining the comparative effectiveness of BAIs for reducing alcohol misuse among college students by utilizing IPD. All BAIs in the sample were delivered individually in person, in group, via mail, or computer/online. All BAIs were considered brief but differed in the content topics covered and levels of personalization (Ray et al., 2014). Of the 24 studies with available IPD (*N* = 12,630 participants) obtained from the original investigators, 19 studies met the following inclusion criteria: (a) at least two-arm randomized trials with a control or comparison group and (b) available outcome measures at baseline and a follow-up within 12 months post intervention (see Table 1 and Fig. 2). From the eligible 19 studies (*N* = 11,655 at baseline), unique interventions or non-randomized cohorts were further excluded, resulting in 10,260 participants from 19 studies at baseline (40.6% men, 73.0% White, 55.3% first-year students). Participants included in the current analysis had outcome data at 1–3 months, 6 months, or 9–12 months post intervention (*N* = 7,704, 38.4% men, 74.7% White, 58.5% first-year students).

**Table 1:** Descriptive statistics of participants by study and follow-up time point

Study	Reference	Follow-up (months)	% White	% Male	% First-year student	Intervention group	*n*
2	White et al. (2008)	1–3	70.6	68.6	62.4	PF	92
						Control	102
4	Cimini et al. (2009)	6	80.2	62	48.6	GMI	159
						Control	154
		9–12	82.1	61.4	47	GMI	149
						Control	136
7.1	Fromme and Corbin (2004)	1–3	76.3	78.4	62.9	GMI	75
						Control	22
7.2	Fromme and Corbin (2004)	1–3	59.1	58.2	40.6	GMI	216
						Control	109
		6	53	48.4	35	GMI	145
						Control	72
8a	Larimer et al. (2007)	9–12	86.6	30	52.4	PF	512
						Control	519
8b	Larimer et al. (2007)	9–12	64.4	39.5	49.9	PF	719
						Control	754
8c	Larimer et al. (2007)	9–12	85	39.8	38.3	PF	127
						Control	147
9	Lee et al. (2009)	1–3	72.7	36.8	100	PF	87
						GMI	84
						MI + PF	84
						Control	82
		6	72.5	36.4	100	PF	82
						GMI	79
						MI + PF	78
						Control	85
10	Baer et al. (2001)	9–12	86.3	45.3	100	MI + PF	150
						Control	157
11	Walters et al. (2007)	1–3	66.7	55.8	100	PF	127
						Control	140
12	Wood et al. (2007)	1–3	92.9	47.4	3.2	MI + PF	75
						Control	79
		6	94.3	46.8	2.8	MI + PF	71
						Control	70
13/14	Murphy et al. (2001, 2004)	1–3	96.3	42.6	38.9	MI + PF	30
						Control	24
		6	94.7	32	22.7	PF	27
						MI + PF	24
						Control	24
		9–12	96.3	42.6	38.9	MI + PF	30
						Control	24
15	LaBrie et al. (2008a)	1–3	56.1	0	100	GMI	139
						Control	98
16	LaBrie et al. (2009)	1–3	57.3	0	100	GMI	153
						Control	126
		6	56.7	0	100	GMI	137
						Control	110
18	Martens et al. (2010)	1–3	88.9	24.9	31.2	PF	90
						Control	99
		6	87.7	22.2	32.7	PF	82
						Control	89
19	LaBrie et al. (2008b)	1–3	66.9	27.8	20.1	PF	435
						Control	489
20	Larimer et al. (2001)	9–12	82.2	48.7	79.4	MI + PF	214
						Control	242
21	Walters et al. (2009)	1–3	85.5	35.5	39.5	PF	60
						MI + PF	73
						Control	67
		6	84.7	37.4	40.5	PF	55
						MI + PF	71
						Control	64
		9–12	84.6	37.2	40.4	PF	56
						MI + PF	69
						Control	63
22	Wood et al. (2010)	9–12	88	43.2	100	MI + PF	228
						Control	240

Follow-up periods were grouped for 1–3 months, 6 months, and 9–12 months in the current study

*MI* + *PF* Individually delivered motivational interviewing intervention with personalized feedback, *PF* stand-alone personalized feedback intervention, *GMI* group motivational interviewing intervention

#### Intervention and Control

Of the 19 studies that met the inclusion criteria, 14 studies were two-arm trials, four studies (studies 9, 13/14, 21) were multi-arm trials, and one study had two subsamples (study 7), resulting in a total of 46 intervention arms: seven motivational interviewing with personalized feedback (MI + PF) interventions, six group motivational interviewing (GMI) interventions, ten stand-alone personalized feedback (PF) interventions, and 23 control groups (see Table 1). All intervention groups received both personalized and general/educational information about alcohol use, including alcohol quantity and frequency and descriptive drinking norms. In addition, all but one study (study 19) provided information on blood alcohol concentration (BAC) and intoxication. Seven studies (studies 4, 9, 10, 15, 16, 18, and 20) further provided information on the biphasic effects of alcohol.

Control groups were waitlist controls (studies 2 and 7.1) or assessment-only controls (studies 7.2, 8a, 8b, 8c, 9, 10, 11, 12, 13/14, 15, 16, 19, 20, 21, and 22). Most of the control groups did not receive any intervention content. However, control participants in studies 4, 15, 16, and 20 received limited information about alcohol use and BAC. Control participants in studies 4, 15, 16, and 18 received general educational information about alcohol-related problems. More details on these intervention groups, coding, and harmonization can be found in previous articles (Mun et al., 2015b; Mun & Ray, 2018; Ray et al., 2014).

### Measures

#### Alcohol Use Quantity

For 16 out of the 19 studies included in the current study, typical weekly drinking was derived from a version of the Daily Drinking Questionnaire (DDQ; Collins et al., 1985), which asks participants to report the number of drinks they consumed on each day of a typical week. We summed the number of drinks consumed across 7 days in a typical week. For studies 15, 16, and 19, baseline values were derived from two single-item measures in which participants were asked to indicate the number of drinks they consumed on a typical drinking occasion, as well as the number of days they consumed alcohol in a typical week. We multiplied these two items to get the total number of drinks consumed in a typical week for these studies. In sum, all 19 studies (studies 2, 4, 7 [7.1 and 7.2], 8a, 8b, 8c, 9, 10, 11, 12, 13, 14, 15, 16, 18, 19, 20, 21, and 22) had typical weekly drinking data at 1–3 months, 6 months, or 9–12 months post intervention (see Fig. 1 for outcome distributions at 1–3 months follow-up).

#### Covariates

Sex (1 = male; 0 = female), first-year student status (1 = first-year student; 0 = non-first-year student), and race (1 = White; 0 = non-white) were included as covariates in all study-specific models, when applicable. In addition, alcohol use quantity (i.e., typical weekly drinking) at baseline was included in all study-specific models as a covariate.

### Analysis Plan

We conducted an IPD meta-analysis to evaluate BAI effects on reducing alcohol use separately at three follow-up periods: 1–3 months, 6 months, and 9–12 months. For the analysis at each follow-up time point, we first derived covariate-adjusted, study-specific intervention effects using appropriate statistical models (i.e., NB or MZIP regression models), one study at a time. The full model used in the analysis was as follows:

$$\begin{array}{l}\mathrm{log}\left({v}_{i}\right)={\beta }_{0}+{\beta }_{1}\cdot (P{F)}_{i}+{\beta }_{2}\cdot (MI+P{F)}_{i}+{\beta }_{3}\cdot (GM{I)}_{i}\\\qquad\qquad+\ {\beta }_{4}\cdot Alcohol\ Use\ at\ {Baseline}_{i}+{\beta}_{5}\cdot Mal{e}_{i}\\\qquad\qquad+\ {\beta }_{6}\cdot Whit{e}_{i}+{\beta }_{7}\cdot {FirstYear}_{i}.\end{array}$$

Since both the NB and MZIP models evaluate the overall mean of the outcome (i.e., ${v}_{i}$), the equation is the same, except that the underlying distribution can be different (NB or ZIP). Because there is no evidence-based recommendation in the literature regarding the conditions in which MZIP is preferred to NB and vice versa, we used MZIP when the observed zero rate exceeded the expected zero rate by greater than 10% (representing moderate to severe zero inflation). For other studies that had little to no zeros, we used NB (see Table 2 and Fig. 1).

**Table 2:** Mean, SD, and zero rate of the number of drinks and the method used

Study	*Mean*	*SD*	Obs. zero rate	Exp. zero rate	Method
*1–3 months (n* = *3,257)*					
2	3.58	4.97	0.27	0.03	MZIP
7.1	15.77	15.34	0.16	0.00	MZIP
7.2	11.30	10.57	0.16	0.00	MZIP
9	9.40	10.33	0.13	0.00	MZIP
11	2.83	5.89	0.67	0.06	MZIP
12	21.64	10.80	0.00	0.00	NB
13/14	18.50	8.77	0.02	0.00	NB
15	3.72	5.32	0.36	0.02	MZIP
16	4.32	6.17	0.37	0.01	MZIP
18	6.26	8.73	0.35	0.00	MZIP
19	7.95	10.03	0.21	0.00	MZIP
21	12.03	12.82	0.09	0.00	NB
*6 months (n* = *1,678)*					
4	18.01	15.76	0.10	0.00	NB
7.2	9.46	10.78	0.18	0.00	MZIP
9	9.83	9.99	0.13	0.00	MZIP
12	20.79	12.77	0.01	0.00	NB
13/14	19.48	10.75	0.00	0.00	NB
16	5.83	8.90	0.49	0.00	MZIP
18	7.44	8.81	0.28	0.00	MZIP
21	11.46	11.78	0.09	0.00	NB
*9–12 months (n* = *4,536)*					
4	18.30	14.94	0.10	0.00	NB
8a	5.57	8.31	0.30	0.00	MZIP
8b	5.00	8.05	0.35	0.01	MZIP
8c	6.21	10.33	0.25	0.00	MZIP
10	12.12	10.32	0.07	0.00	NB
13/14	16.28	8.57	0.00	0.00	NB
20	11.33	13.26	0.18	0.00	MZIP
21	10.53	10.53	0.07	0.00	NB
22	9.34	10.78	0.27	0.00	MZIP

Frequency distributions at 1–3 months are shown in Fig. 1

*MZIP* marginalized zero-inflated Poisson, *NB* negative binomial, *Obs.* Observed, *Exp.* Expected

In both MZIP and NB models, the intervention effect was quantified using a rate ratio (*RR*) or $\mathrm{exp}({\beta }_{1}^{MZIP\;\mathrm{or}\;NB})$ in the MZIP or NB model formulation. Upon obtaining study-specific intervention effect estimates in the first step, we pooled them in the second step in a random-effects meta-analysis model. This analytic approach is called “two-step” or “two-stage” IPD meta-analysis (Simmonds et al., 2015) and has been utilized in prevention research (Jiao et al., 2020; Mun et al., 2022a; White et al., 2015).

All data preparation was conducted using SAS 9.4 (SAS Institute Inc., Cary, NC) and R 4.0.5 (R Core Team, 2021). NB models were run using the R package “MASS” version 7.3 (Venables & Ripley, 2002). To implement the MZIP model, we developed a new R package, “mcount” (version 1.0; Zhou et al., 2022a), which contains the “mzip” function to fit the MZIP model. Meta-analysis was conducted using the R package “metafor” version 2.4 (Viechtbauer, 2010). All statistical tests used a two-sided significance level of 0.05. Annotated computer code in R and data utilized in the current paper are available at Mendeley Data (https://doi.org/10.17632/h2sd5y6fxp.1; Mun et al., 2022b).

## Results

Figure 1 shows the distributions of weekly drinking by study at 1–3 months follow-up and predicted probability densities of the NB and MZIP distributions (shown as overlaid lines). As shown in Fig. 1, the MZIP model accommodated excessive zeros and peaks of nonzero distributions in most studies, whereas the NB model underestimated the frequency of zeros in many studies (e.g., studies 7.1, 7.2, 9, 19) and missed nonzero peaks in most studies. Figure 1 shows that considerable heterogeneity exists in outcome distributions across studies and that the appropriate statistical model for one study may not be appropriate for other studies. Table 2 shows the means, standard deviations, observed and expected zero rates of weekly drinking, and the statistical method used in each study. An *RR* value of 1 indicates no effect, whereas an *RR* less than 1 indicates that participants in the intervention group had more favorable outcomes (i.e., less alcohol use) than controls. Estimated average overall means from the MZIP and NB models at 1–3 months, 6 months, and 9–12 months for each arm per study are provided in the Supplemental Material, Table S1.

**Fig. 3:**
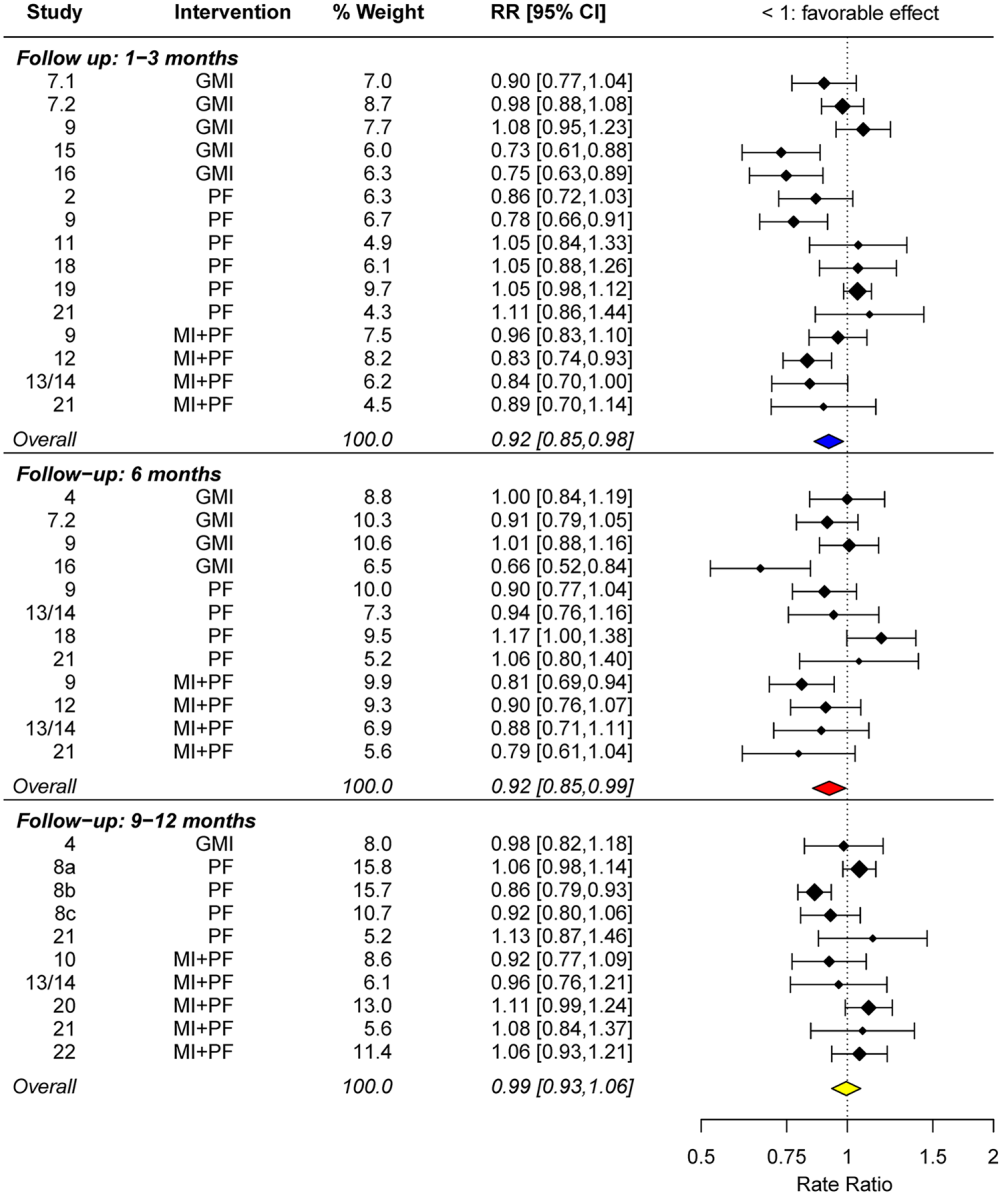
Forest plot of intervention effects at 1–3 months (top), 6 months (middle), and 9–12 months (bottom) follow-up. Note*.* *RR*, rate ratio. MI + PF, individually delivered motivational interviewing intervention with personalized feedback; PF, stand-alone personalized feedback intervention; GMI, group motivational interviewing intervention. *RR* values less than 1 indicate that intervention was beneficial

Figure 3 shows a forest plot at 1–3 months follow-up (top), at 6 months follow-up (middle), and at 9–12 months follow-up (bottom) post intervention. In all analyses, the presence of statistical heterogeneity was observed. *τ*^*2*^, an estimate of the between-study variance around the true overall intervention effect, was 0.01 across all follow-ups. *I*^*2*^, which measures the percentage of the total variability in effect estimates due to true between-study heterogeneity (i.e., *τ*^*2*^) rather than sampling variability, was 69.3%, 53.9%, and 57.3%, at 1–3 months, 6 months, and 9–12 months, respectively. Because this represents moderate to substantial heterogeneity in effect sizes (Higgins & Green, 2011), we performed a random-effects meta-analysis.

The overall effectiveness of BAIs in lowering the mean number of drinks was statistically significant across 15 comparisons at 1–3 months post intervention, with an average *RR* estimate = 0.92 (95% *CI* = [0.85, 0.98]). At 6 months, the statistically significant effect was seen between BAIs and controls in 12 comparisons, with an *RR* = 0.92 (95% *CI* = [0.85, 0.99]). An *RR* of 0.92 can be interpreted as an 8% advantage for BAIs over controls in the mean number of drinks, which was maintained through at least 6 months post intervention. At 9–12 months, however, BAIs did not significantly differ from controls in 10 comparisons, with an *RR* = 0.99 (95% *CI* = [0.93, 1.06]).

Subsequent meta-regression showed that the effect of MI + PF on overall mean drinks translated into a 12% difference (*RR* = 0.88, *p* = .06) at 1–3 months and a 15% difference (*RR* = 0.85, *p* = .02) at 6 months. The intervention effect of GMI was slightly weaker (11% difference, *RR* = 0.89, *p* = .06 at 1–3 months and 9% difference, *RR* = 0.91, *p* = .13 at 6 months) than MI + PF, although their effect sizes were not statistically different from each other. By 9–12 months, all intervention effects were not statistically different from zero (see the Supplemental Material, Table S2).

## Discussion

Based on the body of evidence on BAIs as a whole (Larimer et al., 2021; Tanner-Smith & Lipsey, 2015), BAIs have become a prevailing evidence-based intervention approach on college campuses. Nonetheless, there is room to improve, optimize, and scale up existing BAIs. Toward this goal, it is critical to have a common metric with which any future progress can be measured, and competing interventions can be compared head-to-head. The current study was aimed at providing the best evidence with the most granular data–IPD–and with innovative methods that are appropriate for accommodating outcome distribution heterogeneity, two-step IPD meta-analysis of NB and MZIP models. These findings suggest that BAIs are effective at least for 6 months in reducing the mean number of weekly drinks. The statistically significant advantage for BAIs over control was 8% across the entire population, including nondrinkers at both 1–3 months and 6 months. If a 10% difference is accepted by clinicians as a clinically meaningful change (see Miller & Moyers, 2015), this 8% difference comes close to meeting the threshold for “brief” interventions that typically require less than 2 h to deliver.

Our previous discussion on whether BAIs effectively reduce alcohol consumption for college students has been guarded (Huh et al., 2015). The current findings offer two encouraging advances for prevention research and the IDA literature. First, when nondrinkers are not separately examined using an odds ratio test, the effectiveness of interventions may be better detected and quantified, provided the interventions are not iatrogenic for nondrinkers. There is also evidence that BAIs have protective effects against alcohol-related problems (Huh et al., 2015; Jiao et al., 2020) and driving after 4+/5+ drinks (19% reduction in Odds Ratio; Mun et al., 2022a). Therefore, collectively, evidence suggests the importance of improving and optimizing BAIs for young adults. One area for improvement would be to extend the protective time window of BAIs. Longitudinal analyses from individual studies have shown a typical change pattern where intervention participants respond well initially, followed by a phase of diminishing effect of the intervention. BAIs disrupt and suppress alcohol use but lose their protection slowly over time. Fortunately, recent BAI trials have shown that the effect can last for 12–16 months after the intervention, with some modifications, such as adding other therapeutic elements (e.g., Murphy et al., 2019).

The analytical innovation that we demonstrated in the current study may also help build better consensus about the effectiveness of BAIs among major stakeholders–intervention developers, evaluators, college administrators, college students and their parents, and public health agencies. The progress made at the population level toward reducing high-risk drinking and underage drinking since the 1980s has been remarkable (Hingson et al., 2017; Schulenberg et al., 2019). However, in 2014, several news media outlets published a series of articles featuring a Cochrane Systematic Review (Foxcroft et al., withdrawn, p. 5) that concluded “there are no substantive, meaningful benefits of MI interventions for the prevention of alcohol misuse. Although some significant effects were found, we interpret the effect sizes as being too small … to be of relevance to policy and practice.” This review was withdrawn following a critical review article that pointed out major flaws (Mun et al., 2015a) and was republished in 2016 with updated and corrected data (Foxcroft et al., 2016). As the BAI literature evolves, there is increased awareness that even small to modest effect sizes in individual studies may still be clinically meaningful and lead to a reduction in population-level harms associated with heavy drinking, particularly given the low burden of such approaches.

In connection with IDA, IPD, and evidence synthesis approaches, this study provides a data application example that can be employed by investigators who evaluate individual interventions or those conducting meta-analyses. Discrete count outcomes are common in alcohol research and prevention science research more broadly. By utilizing appropriate statistical models and combining commensurate effect size metrics across multiple studies, it is possible to overcome two major challenges in IDA–lack of overlap in measures and cohorts or samples and lack of available examples demonstrating IDA (Curran et al., 2017). The current study along with publicly available annotated R scripts and data will advance the IDA literature and promote uptake of these methods. Existing methods readily available for individual studies are difficult to implement in IDA studies because modeling becomes complex, often leading to non-convergence, especially when applying one-stage estimation approaches to IPD (see Kontopantelis, 2018; Lin et al., 2022). In other situations, estimates from heterogeneous studies are not obtainable or do not have the same interpretation across studies because of study-level differences in designs or samples (see Jiao et al., 2020 for detailed discussion).

With respect to the current challenge of distribution and sample heterogeneity, it is not feasible to estimate parameters from different underlying distributions simultaneously in the same model. Therefore, a two-step approach to synthesizing IPD while sharing the same interpretation was developed in the current study. Empirical studies comparing one- vs. two-step approaches to the estimation and synthesis of IPD suggest that when focusing on the overall treatment effects for continuous outcomes (Kontopantelis, 2018; Mathew & Nordström, 2010) or binary outcomes (Debray et al., 2012; Lin et al., 2022; Stewart et al., 2012), results from one- and two-step IPD meta-analysis tend to converge. Divergent results are primarily due to different modeling assumptions or specifications, especially under less ideal data conditions (Lin et al., 2022), rather than differences in the one- vs. two-step approach to IPD meta-analysis per se (Burke et al., 2017).

A recent simulation investigation motivated by the challenges of Project INTEGRATE indicated that one- and two-step approaches to IPD converged with adequate coverage and bias for treatment effect estimation in the count portion of the model (Huh et al., 2022a). However, the simulation also revealed that the estimation of the treatment effect on the zero portion had room to improve, especially in more extreme data conditions, regardless of the model or approach (e.g., *k* = 5 studies; *N* = 100 participants per study; or 5% zero vs. 25% zero observations for a “true” hurdle negative binomial model; Huh et al., 2022a; https://ipdmeta.shinyapps.io/IPD_Rshiny/ for an interactive display of simulation outcomes). A major thrust of complex data integration and synthesis such as IDA is to identify mechanisms of behavior change. Therefore, there is a need to continue to develop and implement new models that probe mechanistic mediational paths (e.g., Huh et al., 2022b), subgroups (e.g., Tan et al., 2022a), and person-specific intervention effects.

This study has a number of limitations that can be addressed in the future. First, because there is no evidence-based recommendation in the literature regarding the conditions in which MZIP is preferred to NB and vice versa, we used MZIP when the observed zero rate exceeded the expected zero rate by at least 10%. This seemed like a reasonable decision threshold based on our experience, but a simulation study would be useful to provide more authoritative, evidence-based guidance. Further, more work is needed to disseminate the MZIP model for prevention research. Ongoing theoretical and numerical work suggests that MZIP models generally outperform other competing models, including Gaussian (normal) models, especially when intervention effects on the structural zero (vs. nonzero) and Poisson parts are in the same direction (Zhou et al., 2022b). Second, the marginalized zero-inflated negative binomial model may be possible as a unifying approach to modeling count outcomes. Unfortunately, to our knowledge, there has been no theoretical work on this potentially flexible model for a family of count outcomes in the literature. There is a need to develop flexible and reasonable new models for count outcomes with and without zero inflation and overdispersion as well as new computational tools for easy implementation. Third, the IPD sample in this study was not obtained from a systematic search of the literature. Although a comprehensive or systematic IPD meta-analysis would be very rare due to barriers associated with obtaining IPD, it is uncertain how representative the current IPD sample is relative to a sample that could be systematically searched and obtained. Finally, we had several comparisons that were nested within studies. Although it may not be ideal for analyzing more than one effect size from the same study as if effect sizes were independent, this practice is fairly common in the field. Also, given that most of the trials were two-arm trials, its effect on the inference may be limited.

Analysis of IPD is unequivocally challenging. However, the collective experience of analyzing IPD from Project INTEGRATE has started to yield dividends. Prevention and intervention research share many of the same challenges, and the methods that we introduced in this study may be directly applicable in other studies that pool IPD. While we acknowledge that access to IPD remains a barrier to IDA or IPD meta-analysis, IPD are expected to become more widely available. For example, the National Institute on Alcohol Abuse and Alcoholism (NIAAA) already requires submitting grant-related human subjects data to an NIAAA-sponsored data repository (https://grants.nih.gov/grants/guide/notice-files/NOT-AA-22-011.html). Moreover, the National Institutes of Health will require a data-sharing plan for all grant applications effective January 25, 2023 (Kozlov, 2022). Therefore, new methods for IPD analysis may become more relevant for future studies as IPD availability increases. More generally, data sharing and data reuse have been recognized as promising strategies for discoveries and as ways to improve replicability and public confidence in science. As open science practices (e.g., data sharing) take deeper roots, novel data standardization, harmonization, integration, and synthesis methods may become even more essential to promote discoveries that are rigorous and timely. Finally, we underscore that the innovation of the current work is not limited to data integration and synthesis research. The methods used in the study can be beneficial for individual clinical trials to more precisely measure intervention effects and for multi-site studies that are pre-planned, such as the ABCD study (Volkow et al., 2018). Providing a credible answer to public health problems is a major responsibility for the scientific community, and the rigorous and transparent approach required for IPD analysis may help strengthen scientific practice and public trust in scientific evidence for decision-making.

### Supplementary Information

Below is the link to the electronic supplementary material.

Supplementary file1 (DOCX 48 KB)
