# Supplementary material for: Brief Alcohol Interventions are Effective through 6 Months: Findings from Marginalized Zero-inflated Poisson and Negative Binomial Models in a Two-step IPD Meta-analysis
Source: Prev Sci. 2022 Aug 17;24(8):1608–21. doi: 10.1007/s11121-022-01420-1 (PMC10678823; doi:10.1007/s11121-022-01420-1)
Supplement: Supplementary file 1 — Supplementary file1 (DOCX 48 KB) [file 11121_2022_1420_MOESM1_ESM.docx]

**Supplemental Material**

**Brief Alcohol Interventions are Effective through Six Months: Findings from Marginalized Zero-inflated Poisson and Negative Binomial Models in a Two-step IPD Meta-analysis**

Eun-Young Mun^1^; Zhengyang Zhou^2^; David Huh^3^; Lin Tan^1^; Dateng Li^4^; Emily E. Tanner-Smith^5^; Scott T. Walters^1^;

Mary E. Larimer^6^

*Prevention Science*

**Table S1**

Estimated Overall Means from the MZIP and NB Models at 1-3 Months, 6 Months, and 9-12 Months

|  | 1-3 months | | | |  | 6 months | | | |  | 9-12 months | | | |
| --- | --- | --- | --- | --- | --- | --- | --- | --- | --- | --- | --- | --- | --- | --- |
| Study | Control | GMI | MI+PF | PF |  | Control | GMI | MI+PF | PF |  | Control | GMI | MI+PF | PF |
| 2 | 4.03 | – | – | 3.16 |  | – | – | – | – |  | – | – | – | – |
| 4 | – | – | – | – |  | 17.70 | 19.68* | – | – |  | 18.74 | 19.17* | – | – |
| 7.1 | 13.84 | 14.63 | – | – |  | – | – | – | – |  | – | – | – | – |
| 7.2 | 9.45 | 11.64 | – | – |  | 7.23 | 9.10 | – | – |  | – | – | – | – |
| 8a | – | – | – | – |  | – | – | – | – |  | 5.10 | – | – | 5.41 |
| 8b | – | – | – | – |  | – | – | – | – |  | 5.07 | – | – | 4.31 |
| 8c | – | – | – | – |  | – | – | – | – |  | 6.82 | – | – | 5.38 |
| 9 | 9.27 | 9.78 | 7.45 | 7.78 |  | 10.73 | 10.45 | 7.01 | 9.96 |  | – | – | – | – |
| 10 | – | – | – | – |  | – | – | – | – |  | 12.75 | – | 11.81 | – |
| 11 | 2.45 | – | – | 2.69 |  | – | – | – | – |  | – | – | – | – |
| 12 | 24.27 | – | 19.23 | – |  | 22.46 | – | 19.42 | – |  | – | – | – | – |
| 13/14 | 19.41 | – | 17.88 | – |  | 19.77 | – | 19.55 | 19.41 |  | 15.73 | – | 16.80 | – |
| 15 | 4.52 | 3.15 | – | – |  | – | – | – | – |  | – | – | – | – |
| 16 | 4.28 | 4.00 | – | – |  | 6.41 | 4.87 | – | – |  | – | – | – | – |
| 18 | 5.60 | – | – | 6.14 |  | 6.77 | – | – | 7.65 |  | – | – | – | – |
| 19 | 7.25 | – | – | 8.36 |  | – | – | – | – |  | – | – | – | – |
| 20 | – | – | – | – |  | – | – | – | – |  | 10.98 | – | 10.78 | – |
| 21 | 11.53 | – | 12.28 | 13.11 |  | 12.52 | – | 11.18 | 11.99 |  | 9.81 | – | 11.01 | 11.24 |
| 22 | – | – | – | – |  | – | – | – | – |  | 8.82 | – | 8.92 | – |

*Notes.* MZIP = Marginalized Zero-inflated Poisson. NB = Negative Binomial. * = After removing one extreme outlier for a covariate, alcohol use at baseline. – indicates no outcome data by study design.

**Table S2**

Meta-regression Results

|  | *Est.* | *se* | *z* | *p* | 95% *CI* | | *RR* |
| --- | --- | --- | --- | --- | --- | --- | --- |
| *1-3 months* |  |  |  |  |  |  |  |
| Intercept | −0.133 | 0.071 | −1.881 | 0.060 | −0.271 | 0.006 | 0.876 |
| GMI | 0.017 | 0.093 | 0.181 | 0.856 | −0.166 | 0.199 | 0.891 |
| PF | 0.102 | 0.092 | 1.107 | 0.269 | −0.078 | 0.282 | 0.969 |
| *6 months* |  |  |  |  |  |  |  |
| Intercept | −0.165 | 0.068 | −2.439 | 0.015 | −0.298 | −0.033 | 0.848 |
| GMI | 0.069 | 0.093 | 0.741 | 0.459 | −0.113 | 0.250 | 0.908 |
| PF | 0.173 | 0.096 | 1.810 | 0.070 | −0.014 | 0.360 | 1.008 |
| *9-12 months* | |  |  |  |  |  |  |
| Intercept | 0.031 | 0.053 | 0.579 | 0.563 | −0.073 | 0.134 | 1.031 |
| GMI | −0.048 | 0.135 | −0.353 | 0.724 | −0.313 | 0.217 | 0.983 |
| PF | −0.068 | 0.074 | −0.915 | 0.360 | −0.212 | 0.077 | 0.964 |

*Notes. RR* = Rate Ratio. Intercept = Estimated effect size of MI + PF (i.e., the effect size of a referent intervention group in meta-regression). Estimates of GMI and PF indicate their differences from the MI + PF effect. MI + PF = Individually-delivered Motivational Interviewing Intervention with Personalized Feedback, PF = Stand-alone Personalized Feedback Intervention, GMI = Group Motivational Interviewing Intervention. *RR* values less than 1 indicate that intervention was beneficial.
